# Lip Repositioning Using Scalpel Technique for Management of Excessive Gingival Display Due to Short Upper Lip: A Case Report With One-Year Follow-Up

**DOI:** 10.7759/cureus.115103

**Published:** 2026-08-24

**Authors:** Aman Narang, Mehvish Khan, Nitin Tomar, Mayur Kaushik, Roopse Singh

**Affiliations:** 1 Periodontology &amp; Implantology, Subharti Dental College and Hospital, Swami Vivekanand Subharti University, Meerut, IND

**Keywords:** excessive gingival display, gummy smile, lip repositioning, periodontal plastic surgery, short upper lip, smile esthetics

## Abstract

Excessive gingival display (EGD), commonly referred to as a gummy smile, represents a significant esthetic concern influenced by the interplay of dentogingival, skeletal, and soft tissue factors. Lip repositioning has emerged as a minimally invasive surgical modality aimed at limiting the superior displacement of the upper lip during smiling.

This case report describes the management of a 37-year-old female patient presenting with EGD due to a short upper lip, treated using a conventional scalpel-based lip repositioning technique. Preoperative gingival display of 3 mm was reduced to 0 mm immediately postoperatively. At one-month, six-month, and one-year follow-ups, gingival display was 0.5 mm, 1 mm, and 1.2 mm, respectively, indicating minimal relapse and stable outcomes. Healing was uneventful, and patient satisfaction was high. The findings suggest that lip repositioning is a predictable and effective treatment modality in appropriately selected cases of soft tissue-related EGD.

## Introduction

Smile esthetics plays a pivotal role in overall facial attractiveness and significantly influences an individual’s self-esteem and social interactions. The harmony between the lips, teeth, and gingival framework is essential in creating an esthetically pleasing smile. Among the various components of smile esthetics, the degree of gingival display during smiling is considered a critical parameter. Excessive gingival display (EGD), commonly referred to as a “gummy smile,” is generally perceived as unesthetic when more than 2-3 mm of gingiva is exposed during a full smile [1]. The prevalence of EGD has been reported to be approximately 10% among individuals aged 20-30 years, with a higher occurrence among females [2]. However, the perception of an ideal smile is subjective and may vary depending on cultural, professional, and individual preferences, with studies indicating that both dental professionals and laypersons tend to favor minimal gingival exposure [1,3]. Furthermore, dynamic smile analysis and soft-tissue mobility must be comprehensively appraised, as lip displacement during smiling significantly influences the perceived degree of gingival display [4].

The etiology of EGD is complex and multifactorial, necessitating a comprehensive diagnostic approach for appropriate treatment planning. It can broadly be classified into dentogingival, skeletal, and soft tissue causes. Dentogingival factors include altered passive eruption, gingival enlargement, and short clinical crowns, which result in excessive gingival coverage of the anatomical crown. Skeletal causes, particularly vertical maxillary excess (VME), involve disproportionate downward growth of the maxilla, leading to increased gingival exposure. Vertical facial proportions and skeletal growth patterns play a decisive role in overall smile attractiveness and smile line height [5]. Soft tissue factors, such as hyperactivity of the upper lip elevator muscles or reduced upper lip length, also play a significant role in the manifestation of EGD [6]. In many cases, a combination of these factors may coexist, further complicating diagnosis and treatment selection.

Several classification systems have been proposed to standardize the assessment of smile line and gingival display. Tjan et al. classified smile lines into high, average, and low categories based on the extent of tooth and gingival exposure, with high smile lines demonstrating significant gingival display [7]. Liebart et al. expanded upon this concept by introducing a more detailed classification that incorporates the visibility of the periodontal tissues, ranging from very high smile line (more than 2 mm of gingiva visible) to low smile line (minimal or no gingival display) [8]. These classification systems are valuable in clinical practice, as they aid in diagnosis, communication, and treatment planning.

The management of EGD is highly dependent on accurate identification of the underlying etiology. As emphasized by Garber and Salama, treatment planning should be etiology-driven, as inappropriate selection of therapeutic modality may result in suboptimal esthetic outcomes [6]. For instance, altered passive eruption is effectively managed through crown lengthening procedures [9,10], whereas dentoalveolar extrusion may require orthodontic intrusion. Skeletal discrepancies such as VME often necessitate orthognathic surgical correction, while soft tissue abnormalities such as hyperactive or short upper lip can be addressed using lip repositioning procedures.

Lip repositioning surgery, first described by Rubinstein and Kostianovsky in 1973, has gained increasing attention as a minimally invasive and predictable approach for managing EGD associated with soft tissue etiologies [11]. The procedure involves removal of a strip of mucosa from the maxillary labial vestibule, thereby reducing vestibular depth and restricting the superior displacement of the upper lip during smiling [12]. Compared to more invasive procedures, such as orthognathic surgery, lip repositioning offers advantages including reduced morbidity, shorter surgical time, and improved patient acceptance.

In recent years, several modifications of the conventional lip repositioning technique have been introduced, including myotomy, muscle containment approaches, and laser-assisted procedures, all aimed at improving stability and minimizing relapse [13]. Despite these advancements, the conventional scalpel technique remains widely practiced due to its simplicity, cost-effectiveness, and predictable outcomes.

The present case report aims to demonstrate the clinical effectiveness and one-year stability of a scalpel-based lip repositioning procedure in the management of EGD associated with a short upper lip. By providing longitudinal clinical data and emphasizing the importance of appropriate case selection, this report contributes to the existing body of evidence supporting lip repositioning as a viable treatment modality for EGD.

## Case presentation

A 37-year-old female patient presented with the chief complaint of EGD during smiling. The patient was systemically healthy with no relevant medical history.

**Figure 1:**
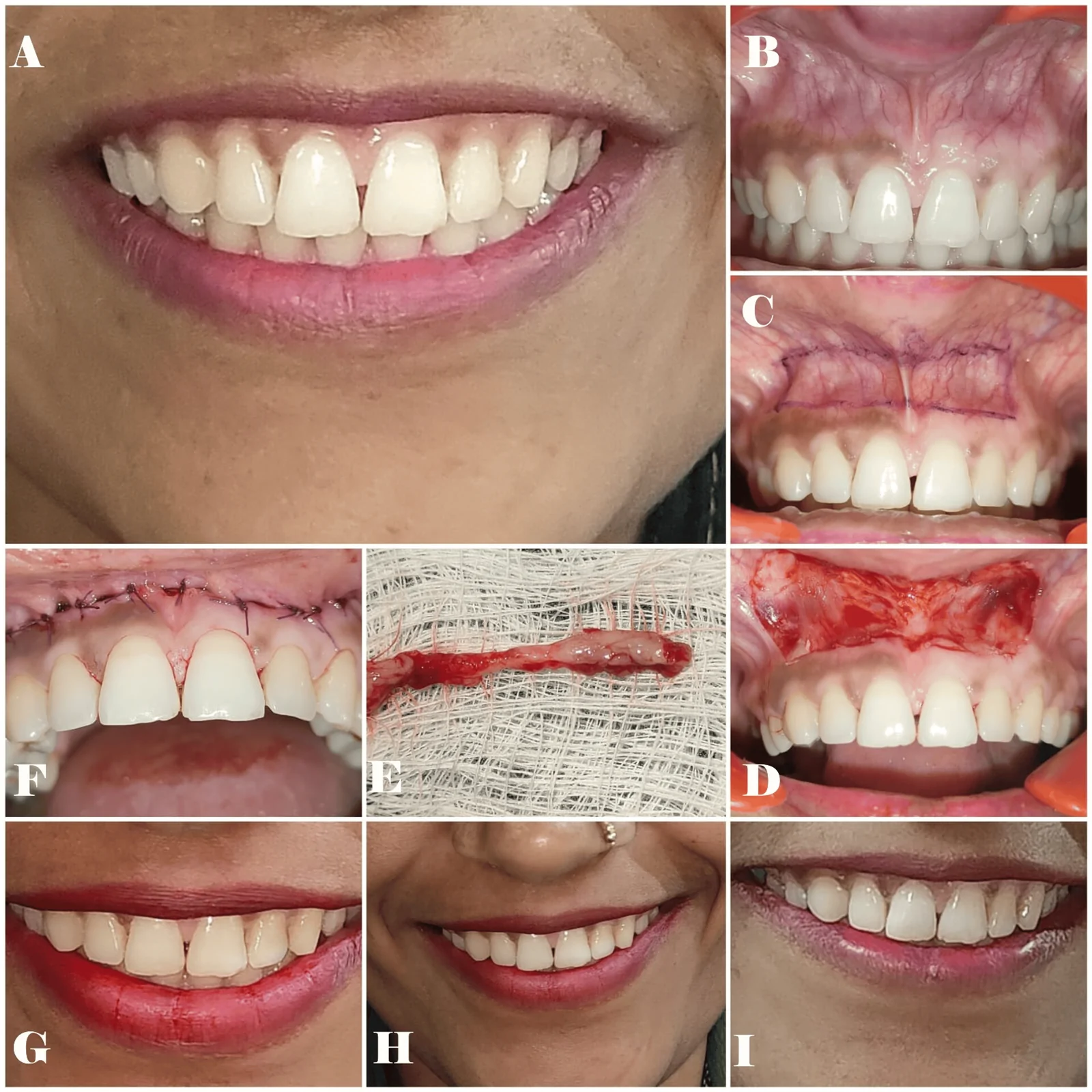
Clinical sequence of lip repositioning surgery for the management of EGD (A) Preoperative extraoral view demonstrating EGD during smiling, characteristic of a gummy smile; (B) Preoperative intraoral view showing the maxillary anterior dentition and gingival architecture prior to surgical intervention; (C) Preoperative surgical marking showing the planned incision design on the maxillary labial mucosa, including the lateral extent and midline reference, corresponding to the area selected for mucosal excision during lip repositioning; (D) Intraoperative view following partial-thickness dissection and removal of the marked mucosal segment, exposing the underlying connective tissue and creating the recipient surfaces for repositioning of the upper lip; (E) Excised strip of labial mucosa obtained following the planned incision, demonstrating the soft-tissue segment removed; (F) Immediate postoperative intraoral view demonstrating coronal repositioning of the upper lip and approximation of the surgical margins with interrupted sutures; (G) One-month postoperative extraoral smile view demonstrating reduction in gingival display and improved tooth-lip relationship following lip repositioning; (H) Six-month postoperative extraoral smile view demonstrating stable reduction in gingival exposure and improved aesthetic appearance following healing; (I) One-year postoperative extraoral smile view showing the final aesthetic outcome, with reduced gingival display and improved harmony between the upper lip and maxillary anterior teeth. EGD: Excessive gingival display

Clinical examination revealed a high smile line with approximately 3 mm of gingival display over the zenith of both the maxillary lateral incisors (Figure 1A). The gingival display was clinically measured in millimeters during maximum posed smiling, using the vertical distance between the inferior border of the upper lip and the gingival margin as the reference points. Because this was a single-case report, repeated measurements for formal intra-observer reliability or calculation of measurement error were not performed. The clinical crown dimensions were within normal limits, and no evidence of altered passive eruption or gingival enlargement was observed (Figure 1B). Based on these findings, a diagnosis of EGD due to a short upper lip was established after ruling out any orthodontic anomalies. Comprehensive proportional and angular facial assessments should be documented to confirm soft-tissue harmony and rule out underlying dentofacial dysmorphology [14].

Surgical procedure

Following aseptic preparation, local anesthesia with a standard concentration of adrenaline was administered. A horizontal incision was placed along the mucogingival junction extending from one maxillary canine to the contralateral canine. A second parallel horizontal incision of equal length was made approximately 10-12 mm apical to the first incision on the labial mucosa. Two vertical incisions were then placed at the ends to connect both horizontal incisions, outlining an elliptical strip of mucosa (Figure 1C).

A partial-thickness flap was elevated, and the delineated mucosal strip was excised (Figures 1D, 1E). The wound margins were approximated and secured using 5-0 PGA/PLA sutures, ensuring tension-free closure (Figure 1F).

Postoperative care

The patient was prescribed analgesics to control postoperative pain and antibiotics for any postoperative complication. Patient was also instructed to limit excessive lip movement. Oral hygiene instructions were provided, and follow-up visits were scheduled to monitor healing.

Result

The postoperative course was uneventful, with satisfactory healing observed at all follow-up intervals. No signs of infection, wound dehiscence, or scar-related complications were noted.

A marked reduction in gingival display was achieved immediately following the surgical intervention, with complete elimination of gingival exposure during smiling. This immediate outcome highlights the effectiveness of the procedure in restricting the superior displacement of the upper lip.

At subsequent follow-up intervals, a gradual yet minimal increase in gingival display was observed: one month - 0.5 mm (Figure 1G); six months - 1 mm (Figure 1H); one year - 1.2 mm (Figure 1I) (Table 1).

**Table 1:** Changes in gingival display following lip repositioning surgery during the one-year follow-up period Gingival display was recorded in millimeters (mm) at baseline (preoperative), immediately postoperatively, and at one-month, six-month, and one-year follow-up intervals. The values demonstrate an immediate reduction in gingival display from 3.0 mm preoperatively to 0 mm immediately postoperatively, with minimal recurrence to 1.2 mm at one year.

Follow-up interval	Gingival display
Preoperative	3.0 mm
Immediate postoperative	0 mm
1 month	0.5 mm
6 months	1.0 mm
1 year	1.2 mm

Despite this slight increase, the gingival display remained within clinically acceptable esthetic limits. The degree of relapse observed was minimal and did not compromise the overall esthetic outcome. 

Patient-reported outcome assessment

Patient satisfaction was assessed at the one-year follow-up using both a Visual Analog Scale (VAS) and a Numerical Rating Scale (NRS). For the VAS, the patient was presented with a 10-cm horizontal line, with the left endpoint designated as 0 (complete dissatisfaction) and the right endpoint as 10 (complete satisfaction). The patient was instructed to mark the point corresponding to her perceived satisfaction, and the distance from the 0-mm endpoint was measured and converted to a score from 0 to 10. The resulting VAS score was 7.7/10. A separate 0-10 NRS was also administered, with 0 representing complete dissatisfaction and 10 representing complete satisfaction; the patient reported a score of 8/10 [15].

**Figure 2:**
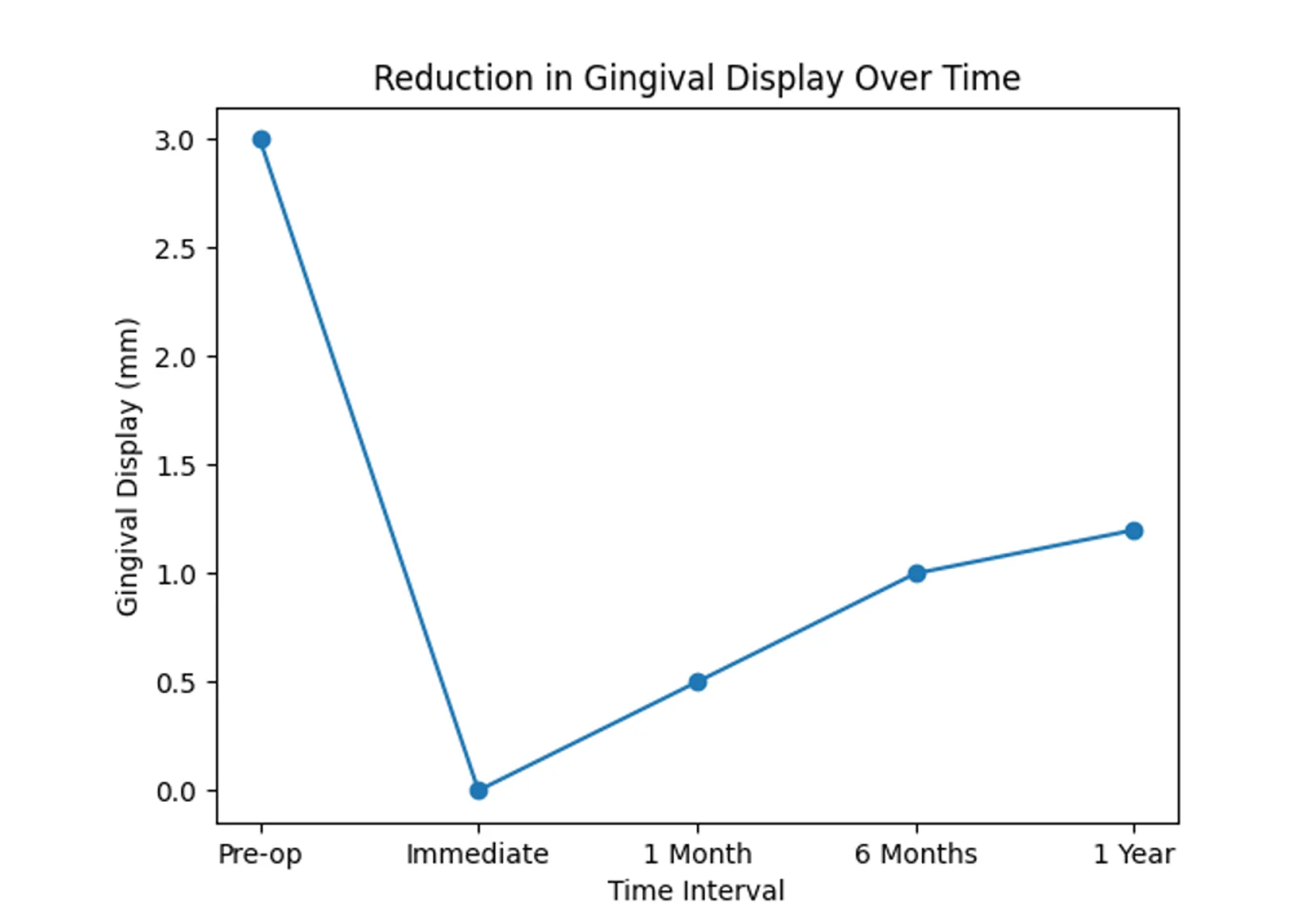
Graphical representation of reduction in gingival display over time

Overall, the results demonstrated immediate and significant esthetic improvement, stable outcomes over a 12-month period, minimal relapse, and high patient acceptance (Figure 2).

## Discussion

Lip repositioning has evolved as a conservative alternative for the management of EGD, particularly in cases where the etiology is confined to soft tissue abnormalities such as a short upper lip or hypermobility. The present case demonstrated a substantial reduction in gingival display from 3 mm to 0 mm immediately postoperatively, followed by a mild relapse to 1.2 mm at one year, which remains within acceptable esthetic limits. A fundamental aspect in the management of EGD is accurate identification of the underlying cause, as treatment outcomes are highly dependent on etiology-based planning. EGD is generally considered clinically significant when gingival exposure exceeds 3 mm during smiling [1], and therefore, treatment must be directed accordingly. Altered passive eruption is best managed by crown lengthening, dentoalveolar extrusion by orthodontic intrusion, VME by orthognathic surgery, and soft tissue abnormalities such as short or hyperactive upper lip by lip repositioning [6]. In the present case, the patient exhibited normal crown proportions along with a short upper lip, thereby eliminating the need for hard tissue correction.

Crown lengthening was not considered an appropriate treatment modality in this case, as it is primarily indicated when excessive gingival coverage results from altered passive eruption or short clinical crowns. The patient demonstrated normal clinical crown dimensions with no evidence of altered passive eruption, indicating that the primary concern was related to lip dynamics rather than gingival excess. Performing crown lengthening under such circumstances would not have corrected the underlying problem and could have led to unfavorable esthetic outcomes, including elongated clinical crowns and compromised smile harmony. This rationale is consistent with existing literature, which emphasizes that crown lengthening is contraindicated when gingival display is attributed to soft tissue imbalance rather than dentogingival discrepancy [10]. The major etiological factors associated with EGD and their commonly considered treatment approaches are summarized in Table 2 [2].

**Table 2:** Major etiologies of EGD and commonly considered treatment approaches EGD: Excessive gingival display; VME: Vertical maxillary excess

Etiological category	Typical clinical feature	Representative treatment approach
Altered passive eruption	Excessive gingival coverage of anatomically normal crowns	Esthetic crown lengthening
Gingival enlargement	Increased gingival tissue covering the clinical crown	Periodontal therapy/gingivectomy or appropriate management of underlying cause
Dentoalveolar extrusion	Excessive vertical position of anterior teeth	Orthodontic intrusion where indicated
VME	Increased lower facial height and generalized gingival display	Orthognathic correction when indicated
Short upper lip	Reduced upper-lip length contributing to gingival exposure	Lip repositioning in appropriately selected cases
Hypermobile/hyperactive upper lip	Excessive superior movement of the upper lip during smiling	Lip repositioning, muscle-directed procedures, or other appropriate modalities
Multifactorial EGD	Combination of two or more etiologies	Individualized, combined treatment

Lip repositioning is generally indicated in patients presenting with mild to moderate gingival display, typically ranging from 2 to 6 mm, particularly when the etiology involves a short or hyperactive upper lip and when an adequate width of attached gingiva is present [11]. It is especially suitable for patients seeking minimally invasive alternatives to more aggressive surgical procedures. Studies have reported that lip repositioning can achieve an average reduction in gingival display of approximately 3-4 mm, making it an effective option in appropriately selected cases [12]. However, the effectiveness of this technique is limited in cases involving skeletal discrepancies. When VME exceeds approximately 6 mm, skeletal correction becomes more appropriate, and lip repositioning may not yield satisfactory results. Furthermore, in cases where VME exceeds 8 mm, lip repositioning is generally contraindicated, and orthognathic procedures such as Le Fort I osteotomy are required to reposition the maxilla and address the underlying skeletal deformity [13]. Therefore, lip repositioning should be regarded as a soft tissue camouflage procedure rather than a substitute for skeletal correction.

A gradual relapse pattern was observed in the present case, with gingival display increasing from 0 mm immediately postoperatively to 1.2 mm at one year. This finding is consistent with existing literature, which frequently reports mild relapse following lip repositioning procedures [9,12]. The relapse is primarily attributed to factors such as muscle reattachment, functional adaptation, and the elastic recoil of soft tissues. Additionally, the conventional technique does not involve muscle detachment, which may contribute to partial recurrence over time. Various modifications to the conventional technique, including myotomy, muscle containment approaches, and lip stabilization procedures such as the LipStaT technique, have been proposed to minimize relapse [16]. While these modifications may enhance stability, they are often associated with increased surgical complexity and technique sensitivity. Despite the occurrence of relapse, most studies have concluded that the degree of recurrence is partial and remains clinically acceptable, as was observed in the present case.

When compared to other treatment modalities, lip repositioning offers a favorable balance between effectiveness, invasiveness, and patient acceptance. Orthognathic surgery, although definitive in cases of skeletal discrepancy, is highly invasive and associated with increased morbidity, making it unsuitable for mild cases [13]. Crown lengthening is effective in cases of altered passive eruption but fails to address lip dynamics and may compromise esthetics if misapplied [10]. Botulinum toxin injections provide a minimally invasive option; however, their effects are temporary, typically lasting three to six months, and require repeated administration [17]. Laser-assisted lip repositioning has been reported to offer advantages such as reduced intraoperative bleeding and faster healing, although long-term outcomes are comparable to those achieved with the scalpel technique [18]. Modified lip repositioning techniques, including myotomy and LipStaT, may reduce relapse but involve greater surgical complexity [13]. In this context, the conventional scalpel technique remains a reliable, cost-effective, and widely applicable option in clinical practice.

The major strength of this case report is the appropriate selection of a patient with EGD primarily attributable to a short upper lip, with normal clinical crown dimensions and no evidence of altered passive eruption or gingival enlargement. The study provides longitudinal clinical observations over a one-year period, allowing assessment of both the immediate treatment response and subsequent relapse. Objective measurements of gingival display were recorded, demonstrating a reduction from 3 mm preoperatively to 1.2 mm at one year. The inclusion of multiple follow-up intervals provides useful information regarding the stability of the conventional scalpel technique. In addition, the absence of postoperative complications and the inclusion of a patient-reported VAS satisfaction score of 7.7 and NRS score of 8 provide both clinical and patient-centered evidence of treatment success.

The present case report has several limitations that should be considered when interpreting the findings. First, the study represents a single-case report, and therefore the findings cannot be generalized to the broader population of patients with EGD. Individual variations in lip anatomy, muscle activity, gingival display, and healing response may influence the clinical outcome. Second, the absence of a control group or comparison with alternative treatment modalities limits the ability to determine whether the observed improvement and stability are superior to other approaches for the management of EGD. Another limitation is the limited number of clinical photographs documenting the preoperative and postoperative stages, particularly photographs demonstrating the actual measurement of gingival display. Longer follow-up periods involving multiple patients would be necessary to determine whether the minimal relapse observed in this case is maintained over several years. In addition, formal quantitative facial measurements and cephalometric assessment were not documented in this case. Also, additional parameters such as upper-lip length at rest and during smiling, incisor display at rest, lip mobility, and vestibular depth were missing which would have provided a more comprehensive assessment.

## Conclusions

Within the limitations of a single case report, lip repositioning using a conventional scalpel technique demonstrated predictable and satisfactory outcomes in the management of EGD associated with a short upper lip. The procedure resulted in immediate elimination of gingival display, with only minimal relapse observed over a one-year follow-up period. Importantly, the final gingival display remained within acceptable esthetic limits, contributing to a high level of patient satisfaction. Lip repositioning offers several advantages, including minimal invasiveness, reduced morbidity, and relatively simple surgical execution. It represents a viable and effective alternative to more invasive surgical interventions in appropriately selected cases. Long-term stability, ease of execution, and favorable patient-reported outcomes support its continued use in periodontal plastic surgery. However, further studies with larger sample sizes and extended follow-up periods are required to establish definitive evidence regarding long-term predictability.
